# “Sex” and body region effects on bone mineralization in male pigs

**DOI:** 10.5194/aab-63-103-2020

**Published:** 2020-04-02

**Authors:** Maren Bernau, Juliane Schrott, Sebastian Schwanitz, Lena Sophie Kreuzer, Armin Manfred Scholz

**Affiliations:** 1 Livestock Center Oberschleissheim of the Veterinary Faculty, Ludwig-Maximilians-Universität München, St. Hubertusstrasse 12, 85764 Oberschleissheim, Germany; 2 Faculty of Agriculture, Economics and Management, Nuertingen-Geislingen University, Neckarsteige 6–10, 72622 Nürtingen, Germany

## Abstract

Lameness in pigs is one of the major reasons for culling and early losses in pigs. This can be linked to osteoporosis due to pathologic alterations in bone mineral density (BMD) or bone mineral content (BMC) and may also be linked to the sex. Dealing with the ban on piglet castration without anaesthesia in Germany 2021, we have three male “sex” types: entire boars (EB), immunocastrated boars (IB), and surgically castrated boars (SB). The hypothesis of the present study is that BMC or BMD varies between different male sex types. If sex has an effect on bone mineralization (BMC or BMD) and if this affects leg health, it could result in more lameness and problems during fattening in the negatively affected sex type. The present study evaluated bone mineralization (in terms of BMD and BMC) and body composition traits using dual-energy X-ray absorptiometry (DXA) three times during growth at 30, 50, and 90 kg live body weight. Nine body regions were analysed for bone mineral traits and compared for different male sex types and the fattening season. Significant differences were found regarding BMD (and BMC) among EB, IB, and SB for whole-body BMD (BMC). Additionally significant differences were found in the front and lower hind limbs, where SB showed a significantly higher BMD compared to EB, with IB in between. Additionally regional differences were detected among the groups. Further studies are needed to evaluate the effect of these differences in bone mineralization on leg health.

## 1 Introduction

Lameness in pigs is one of the major reasons for culling and early loss in pigs (Friendship et al., 1986; Johnston et al., 1987; Fukawa and Kusuhara, 2000; Stalder et al., 2004; Pluym et al., 2011). Several reasons for lameness are named in the literature with regard to several aspects and contexts (Stalder et al., 2004; Tarrés et al., 2006a, b; Hoge and Bates, 2011).

In human medicine, bone mineral density (BMD) or bone mineral content (BMC) are of major interest in managing osteoporosis (Ryan, 1997). To monitor BMC or BMD dual-energy X-ray absorptiometry (DXA) is widely used (Carter et al., 1992; Ryan, 1997; Tothill, 1995; Tothill and Hannan, 2002, 2007). This method is based on the attenuation of two X-ray beams at different energy levels. Based on the tissue-dependent attenuation factor, the software helps to calculate BMD and BMC in the whole body or in various defined body parts (see Pietrobelli et al., 1996).

DXA has been successfully used to evaluate BMC or BMD in the live pig. Using DXA, beside bone mineral data, soft lean and fat tissue can be determined as well. DXA measurements resulted in comparable data with chemical analysis in terms of body composition and bone mineral content in pigs (Mitchell et al., 1996; Scholz et al., 2002, 2004).

Differences regarding BMC or BMD based on sex or the hormonal condition of animals have been detected and discussed previously for sheep (Arens et al., 2007). Piglet castration without anaesthesia will be banned at the end of 2020 in Germany. Therefore, new male sex types have to be considered: entire boars (EB), immunocastrated boars (IB), and surgically castrated boars (SB). The hypothesis of the present study is that BMC or BMD varies between different male pig sex types. If a sex type has a negative effect on bone mineralization (BMC or BMD), it could result in more lameness and problems during fattening in the male sex type negatively affected.

## 2 Material and methods

### 2.1 Animals

A total number of 101 male pigs was used in this study, consisting of 34 EB, 34 IB, and 33 SB. They were examined in three experimental groups, and each sex type was equally distributed to each group (Table 1). The average age among sex groups differed only slightly, with EB = 75.9±1.0 d, SB = 76.2±1.2 d, and IB = 76.9±1.0 d at first examination (scan 30). This difference did not change during the experiment because the following examination always took place 35 d (scan 50) or 77 d (scan 90) after first examination.

**Table 1:** Description of the experimental animals, divided into three experimental groups (I–III).

Group	Born	Slaughtered	Number	Number	Number
			of EB	of IB	of SB
I	Jul	Dec	13	12	12
II	Sep	Feb	11	11	11
III	Jan	Jul	10	11	10

EB: entire boars. IB: immunocastrated boars. SB: surgically castrated boars.

All animals were kept according to the German national animal welfare regulations (Germany, 2016, 2017). The animal experiment was approved by the District Government of Upper Bavaria (registry numbers 55.2-1-54-2532.2-12-13).

The animals were born and raised on a conventional pig farm. They were F1 crossbred offsprings of German Landrace sows mated with Piétrain boars. Animals were housed in groups as outlined in Table 1 in an outdoor climate barn (individual space > 1.46 m${}^{2}$) and were fed ad libitum with a diet containing 15 MJ ME kg${}^{-1}$. IB were injected twice with a gonadotropin-releasing factor analogon (Improvac™, Pfizer): the first injection was given at an age of 76 d and the second injection at an age of 138 d.

### 2.2 Dual-energy X-ray absorptiometry (DXA)

A GE Lunar iDXA (General Electric Company) was used to perform the measurements for bone mineral content (BMC, g) and bone mineral density (BMD, g cm${}^{-2}$) during a whole-body measurement. The animals were bedded in a prone position with their front limbs flexed and hind limbs extended. The whole-body software mode “dick” (German for “thick”) was used for all examinations. Pigs were examined three times during growth: firstly at approximately 30 kg live body weight (29.84±7.26 kg; scan 30), secondly at approximately 50 kg live body weight (53.59±8.91 kg; scan 50), and thirdly at approximately 90 kg live body weight (92.33±10.59 kg; scan 90).

**Figure 1:**
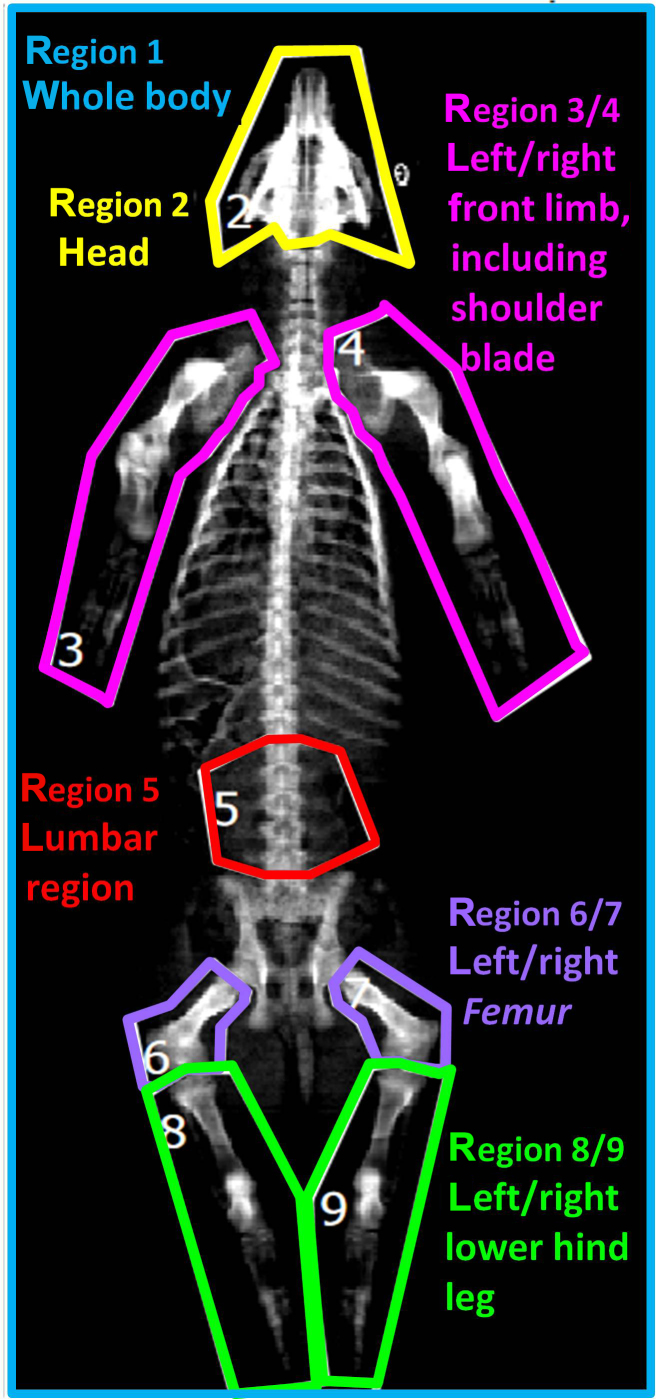
Defined body regions in the two-dimensional DXA image.

Based on the two-dimensional DXA image, a regional analysis was performed by defining nine body regions as demonstrated in Fig. 1: region 1 (R1) – whole body; region 2 (R2) – head; region 3/4 (R3/R4) – left/right front limb including shoulder blade; region 5 (R5) – lumbar region; region 6/7 (R6/R7) – left/right femur; region 8/9 (R8/R9) – left/right lower hind leg.

Beside the BMD (g cm${}^{-2}$) and BMC (g) of each region, soft lean tissue (kg; %), fat tissue (kg; %), and total tissue (kg) were evaluated for the whole body (region 1).

### 2.3 Statistical analysis

A mixed model procedure using SAS 9.3 software was used for statistical analysis. Two different REML (restricted maximum likelihood estimation) models were applied. Model 1 covers the bone mineral traits, i.e. bone mineral density (BMD) and bone mineral content (BMC) with the fixed effects: sex, group, scan time, DXA region, and all possible two-way interactions, while the animal was treated as repeated effect (Tables 3–6). Model 2 covers all DXA traits and weight as well as age. The fixed effects for model 2 are defined as sex, group, scan time, sex × scan time, sex × group, and group × scan time, while like in model 1 the animal was treated as random, and repeated effect (Tables 2, 7).

The estimated LSM (least squares means) were t-tested after a Tukey–Kramer adjustment. The significance level was set to p<0.05 in all cases.

**Table 2:** “Sex” effect on bone mineralization. The table represents least squares means (± standard errors of estimation) of bone mineral density (BMD) and bone mineral content (BMC) for the nine body regions (R1–R9) divided into the three different scans at 30, 50, and 90 kg body weight (30, 50, and 90) and the different sex types (EB, IB, and SB). Different superscripts represent significant (p<0.05) differences within scan, column, and trait.

Scan	Trait	Group	R1	R2	R3	R4	R5	R6	R7	R8	R9
30	BMD	EB	0.538 ± 0.009	1.117 ± 0.021	0.565 ± 0.011	0.556 ± 0.011	0.451 ± 0.011	0.647 ± 0.013	0.638 ± 0.013	0.461 ± 0.008	0.461 ± 0.008
	(g cm${}^{-2}$)	IB	0.551 ± 0.009	1.144 ± 0.021	0.568 ± 0.011	0.561 ± 0.011	0.466 ± 0.011	0.658 ± 0.013	0.653 ± 0.013	0.466 ± 0.008	0.468 ± 0.008
		SB	0.559 ± 0.009	1.140 ± 0.021	0.579 ± 0.012	0.582 ± 0.011	0.459 ± 0.011	0.674 ± 0.013	0.676 ± 0.013	0.470 ± 0.008	0.478 ± 0.009
	BMC	EB	554.40 ± 25.75	118.23 ± 4.84	61.78 ± 3.45	60.84 ± 3.39	17.89 ± 1.14	17.81 ± 1.02	17.62 ± 1.07	38.25 ± 1.83	38.15 ± 1.90
	(g)	IB	574.81 ± 25.49	122.23 ± 4.79	64.80 ± 3.41	63.84 ± 3.36	18.55 ± 1.13	18.60 ± 1.01	18.61 ± 1.06	39.43 ± 1.82	39.09 ± 1.89
		SB	583.49 ± 26.20	123.51 ± 4.92	64.54 ± 3.49	64.55 ± 3.43	18.10 ± 1.17	18.81 ± 1.02	19.10 ± 1.08	39.28 ± 1.86	39.33 ± 1.93
50	BMD	EB	0.713 ± 0.009	1.459 ± 0.021	0.770 ± 0.011	0.752 ± 0.011	0.636 ± 0.010	0.840 ± 0.012	0.834 ± 0.013${}^{a}$	0.615 ± 0.008	0.608 ± 0.008
	(g cm${}^{-2}$)	IB	0.731 ± 0.009	1.498 ± 0.021	0.787 ± 0.011	0.776 ± 0.011	0.654 ± 0.010	0.873 ± 0.012	0.858 ± 0.013${}^{a,b}$	0.624 ± 0.008	0.626 ± 0.008
		SB	0.748 ± 0.009	1.507 ± 0.021	0.801 ± 0.011	0.794 ± 0.011	0.665 ± 0.011	0.895 ± 0.013	0.892 ± 0.013${}^{b}$	0.645 ± 0.008	0.643 ± 0.008
	BMC	EB	1021.48 ± 25.48	206.17 ± 4.78	118.51 ± 3.41	118.83 ± 3.35	36.34 ± 1.13	35.13 ± 1.01	35.19 ± 1.05	72.12 ± 1.80	71.51 ± 1.88
	(g)	IB	1062.41 ± 25.36	212.72 ± 4.76	125.82 ± 3.41	124.31 ± 3.34	37.72 ± 1.12	37.48 ± 1.01	37.32 ± 1.05	75.32 ± 1.80	75.13 ± 1.87
		SB	1055.95 ± 25.79	207.67 ± 4.84	125.33 ± 3.45	123.61 ± 3.39	37.23 ± 1.14	37.40 ± 1.02	37.86 ± 1.07	75.36 ± 1.83	74.87 ± 1.90
90	BMD	EB	0.958 ± 0.009${}^{a}$	1.922 ± 0.021	1.000 ± 0.011${}^{a}$	0.994 ± 0.011${}^{a}$	0.887 ± 0.010	1.115 ± 0.012${}^{a}$	1.109 ± 0.013${}^{a}$	0.816 ± 0.008${}^{a}$	0.819 ± 0.008${}^{a}$
	(g cm${}^{-2}$)	IB	0.974 ± 0.009${}^{a,b}$	1.986 ± 0.021	1.030 ± 0.010${}^{a,b}$	1.032 ± 0.011${}^{a,b}$	0.907 ± 0.010	1.165 ± 0.012${}^{a,b}$	1.156 ± 0.013${}^{a,b}$	0.834 ± 0.008${}^{a,b}$	0.843 ± 0.008${}^{a,b}$
		SB	0.994 ± 0.007${}^{b}$	1.988 ± 0.021	1.062 ± 0.011${}^{b}$	1.061 ± 0.011${}^{b}$	0.920 ± 0.011	1.174 ± 0.013${}^{b}$	1.188 ± 0.011${}^{b}$	0.859 ± 0.008${}^{b}$	0.865 ± 0.008${}^{b}$
	BMC	EB	1804.44 ± 25.48	353.51 ± 4.81	210.33 ± 3.40	208.88 ± 3.35	67.84 ± 1.13	64.80 ± 1.01	64.28 ± 1.05	125.97 ± 1.80	125.29 ± 1.88
	(g)	IB	1866.03 ± 25.36	363.92 ± 4.76	222.91 ± 3.39	221.80 ± 3.34	71.04 ± 1.12	68.80 ± 1.01	68.49 ± 1.05	132.30 ± 1.80	132.07 ± 1.87
		SB	1832.76 ± 25.79	355.99 ± 4.84	222.31 ± 3.45	218.29 ± 3.39	69.69 ± 1.14	67.10 ± 1.02	67.98 ± 1.07	129.23 ± 1.83	128.49 ± 1.89

## 3 Results

### 3.1 Interaction sex × scan

Significant differences between the different body regions were detected. Table 2 represents the data for all nine body regions (R1–R9) divided into the different scan times (30, 50, and 90) and the different sex types (EB, IB, and SB). Both, BMC and BMD show an increase from scan to scan with differences among (1) body parts and (2) sex types.

1. *Body parts*: the highest BMD in all scans was found in the head (R2). BMD records showed the same ranking within the limbs throughout the three scans at 30, 50, and 90 kg body weight with the highest BMD in R6/R7 (femur) followed by R3/R4 (front limb), and R8/R9 (lower hind limbs). In contrast, the highest BMC was found in R3/R4 (front limb) followed by R8/R9 (lower hind limbs) and R6/R7 (femur).
2. *Sex types*: no significant differences were detected regarding the whole-body (R1) records for BMD and BMC at the 30 kg scan. Significant differences among sex types were found for the front limbs (R4, BMD), and the femur (R7, BMD). At 50 kg significant differences were detected for R7 between SB and EB, with IB in between. SB showed the highest BMD values in all regions. At 90 kg, significant differences were detected for whole-body BMD (R1), with SB having the highest and EB having the lowest value and IB being in between. In all limb regions (R3/R4, R6/R7, R8/R9), EB showed the lowest value, with significant differences to SB.

**Table 3:** BMD and BMC depending on body region (LSM ± SEE, standard error of estimation). Different superscripts represent significant (p<0.05) differences within column.

Region	BMD (g cm${}^{-2}$)	BMC (g)
Whole body (R1)	0.7516${}^{a}$	1151.69${}^{a}$
Head (R2)	1.5314${}^{b}$	229.71${}^{b}$
Left front (R3)	0.7959${}^{c}$	135.39${}^{c}$
Right front (R4)	0.7904${}^{c}$	134.12${}^{c}$
Lumbar region (R5)	0.6725${}^{d}$	41.72${}^{d}$
Left femur (R6)	0.8945${}^{e}$	40.75${}^{d}$
Right femur (R7)	0.8903${}^{e}$	40.83${}^{d}$
Left lower hind leg (R8)	0.6437${}^{f}$	80.93${}^{e}$
Right lower hind leg (R9)	0.6463${}^{f}$	80.63${}^{e}$
±SEE (all traits)	±0.004	±2.93

Table 3 represents the data for BMD and BMC depending on the body region among all sex types. Head (R2) showed the highest BMD; the limbs (front and hind leg) showed similar results compared between left and right body side.

The variance analysis shows significant effects for all fixed effects and all possible two-way interactions for the bone mineral traits used with the exception of sex × scan interaction for BMC (Table 4).

**Table 4:** Variance analysis table for bone mineral traits (BMD/BMC).

			BMD		BMC	
Effect	No. DF (degrees of freedom)	Den. DF	F value	Pr >F	F value	Pr >F
Sex (GE)	2	2626	74.71	< 0.0001	7.29	0.0007
Group (GR)	2	2626	23.37	< 0.0001	12.56	< 0.0001
Scan number (scan)	2	2626	10 787.4	< 0.0001	4838.60	< 0.0001
GE × GR	4	2626	17.10	< 0.0001	5.86	0.0001
GR × scan	4	2626	8.70	< 0.0001	4.72	0.0009
GE × scan	4	2626	3.53	0.0070	0.59	0.6727
Region	8	2626	4708.73	< 0.0001	14 590.8	< 0.0001
Scan × region	16	2626	99.46	< 0.0001	1466.94	< 0.0001

### 3.2 Differences between sex types

Only small differences were detected for weight (total tissue mass by DXA: sum of fat mass (g), soft lean tissue mass (g), and bone mineral content, (g)) among the different sex types (see Table 7 – and explanation for body composition below). Table 5 represents the data for BMD and BMC among the average of all measurements, divided for the different sex types. Significant differences were observed between EB and the other sex types for BMD and BMC and between all sex types regarding BMD, with SB having the highest BMD and EB the lowest BMC and BMD.

**Table 5:** BMD (g cm${}^{-2}$) and BMC (g) depending on sex (LSM ± SEE) – average of nine measurements (body regions). Different superscripts represent significant (p<0.05) differences within column.

Sex	BMD (LSM ± SEE)	BMC (LSM ± SEE)
EB	0.825 ± 0.0023${}^{a}$	209.98 ± 1.69${}^{a}$
IB	0.849 ± 0.0023${}^{b}$	218.87 ± 1.68${}^{b}$
SB	0.865 ± 0.0023${}^{c}$	216.41 ± 1.71${}^{b}$

Table 6 represents differences between the sex types divided for the different scan times, both for BMD and BMC. Significant differences can be detected at the 30 kg measurement between EB and SB (BMD) and from the 50 kg measurement on between EB and the other two (BMD). EB has the lowest BMD and BMC for all three measurements. For BMC, significant differences can first be detected at the 90 kg measurement, with EB and IB showing significant differences and SB being in between.

**Table 6:** BMD and BMC depending on sex and scan time (LSM ± SEE) – average of nine measurements (body regions). Different superscripts within trait characterize significant differences (p<0.05).

	BMD (g cm${}^{-2}$)		
Scan	EB	IB	SB
30	0.604 ± 0.004${}^{h}$	0.619 ± 0.004${}^{g,h}$	0.629 ± 0.004${}^{g}$
50	0.803 ± 0.004${}^{f}$	0.815 ± 0.004${}^{e}$	0.843 ± 0.004${}^{d}$
90	1.069 ± 0.004${}^{c}$	1.103 ± 0.004${}^{b}$	1.123 ± 0.004${}^{a}$
	BMC (g)		
Scan	EB	IB	SB
30	103.14 ± 2.98${}^{d}$	108.15 ± 2.94${}^{d}$	108.45 ± 3.05${}^{d}$
50	190.60 ± 2.88${}^{c}$	198.76 ± 2.88${}^{c}$	197.24 ± 2.92${}^{c}$
90	336.19 ± 2.89${}^{b}$	349.72 ± 2.88${}^{a}$	343.53 ± 2.92${}^{a,b}$

**Table 7:** Description of the different body composition variables of the three scan times (30, 50, and 90), divided into the different sex types (EB, IB, and SB) and examination groups (I, II, and III). Different superscripts represent significant (p<0.05) differences within trait.

		Fat (%)			Fat (kg)			Soft lean tissue (kg)			Total tissue (kg)		
Variable	Scan	30	50	90	30	50	90	30	50	90	30	50	90
sex	EB	8.41 ± 0.24${}^{d,e}$	8.87 ± 0.24${}^{d,e}$	11.20 ± 0.24${}^{b,c}$	2.28 ± 0.28 ${}^{d}$	4.49 ± 0.28 ${}^{c}$	9.83 ± 0.28 ${}^{b}$	24.65 ± 0.94${}^{c}$	45.38 ± 0.94${}^{b}$	77.13 ± 0.94${}^{a}$	27.56 ± 1.20${}^{c}$	50.90 ± 1.19${}^{b}$	88.76 ± 1.19${}^{a}$
	IB	8.19 ± 0.24${}^{e}$	9.05 ± 0.24${}^{d}$	12.14 ± 0.24${}^{b}$	2.24 ± 0.28${}^{d}$	4.73 ± 0.28${}^{c}$	10.83 ± 0.28${}^{b}$	25.03 ± 0.93${}^{c}$	46.13 ± 0.93${}^{b}$	76.35 ± 0.93${}^{a}$	27.74 ± 1.19${}^{c}$	51.93 ± 1.19${}^{b}$	89.05 ± 1.19${}^{a}$
	SB	9.02 ± 0.25${}^{d,e}$	10.32 ± 0.25${}^{c}$	15.63 ± 0.25${}^{a}$	2.55 ± 0.29${}^{d}$	5.26 ± 0.29${}^{c}$	13.92 ± 0.29${}^{a}$	25.44 ± 0.95${}^{c}$	45.82 ± 0.95${}^{b}$	74.72 ± 0.95${}^{a}$	28.83 ± 1.22${}^{c}$	52.13 ± 1.20${}^{b}$	90.47 ± 1.21${}^{a}$
Group	I	8.05 ± 0.24${}^{e,f}$	9.69 ± 0.23${}^{c,d}$	13.74 ± 0.23${}^{a}$	2.39 ± 0.27${}^{e}$	5.38 ± 0.27${}^{c}$	12.84 ± 0.27${}^{a}$	26.97 ± 0.90${}^{e}$	49.75 ± 0.90${}^{c}$	79.25 ± 0.89${}^{a}$	29.97 ± 1.14${}^{e}$	56.23 ± 1.14${}^{c}$	93.92 ± 1.14${}^{a}$
	II	8.88 ± 0.25${}^{d,e}$	7.95 ± 0.25${}^{f}$	12.12 ± 0.25${}^{b}$	2.44 ± 0.09${}^{e}$	3.95 ± 0.29${}^{d}$	10.91 ± 0.29${}^{b}$	24.63 ± 0.95${}^{e}$	45.46 ± 0.95${}^{d}$	77.43 ± 0.95${}^{a}$	27.84 ± 1.20${}^{e}$	50.45 ± 1.20${}^{d}$	90.23 ± 1.20${}^{a}$
	III	8.70 ± 0.25${}^{d,e,f}$	10.70 ± 0.25${}^{c}$	13.10 ± 0.25${}^{a,b}$	2.25 ± 0.29${}^{e}$	5.15 ± 0.29${}^{c,d}$	10.83 ± 0.29${}^{b}$	23.51 ± 0.98${}^{e}$	42.13 ± 0.98${}^{d}$	71.52 ± 0.98${}^{b}$	26.32 ± 1.24${}^{e}$	48.28 ± 1.24${}^{d}$	84.12 ± 1.24${}^{b}$

Table 7 presents the whole-body composition variables for all three scans (30, 50, and 90) divided into sex types (EB, IB, and SB) and examination groups (I, II, and III). Differences among (1) sex types and (2) group effects were observed.

1. *Sex type*: no obvious changes were detected over the three scans. SB showed the significantly highest amount and percentage of whole-body fat tissue (kg, %) over all three scans. EB and IB showed similar fat tissue records (kg, %). No differences were detected regarding soft lean tissue (kg) or bone mineral tissue (kg).
2. *Group effect:* At scan 50, group II showed the lowest amount and percentage of fat tissue (%, kg). At scan 90 between group II and III no significant differences could be detected any longer for fat tissue (kg). Regarding soft lean tissue (kg), significant differences were detected at scan 90, where group I and II differ significantly from group III. No differences were detected for bone mineral tissue either between the sex types or between the groups.

## 4 Discussion

The results of this study underline the hypothesis that BMC or BMD varies between different male sex types (Table 5). Additionally this study indicates differences in BMD among the body regions (Tables 2 and 3) and that the rearing season might have an influence on bone mineralization (and body composition) parameters (Table 7). All evaluated parameters increased during the scans, which is in line with the results of Ryan et al. (2011). Three major differences were detected comparing data of EB, IB and SB and the three different groups (I, II, and III).

### 4.1 Discussion regional differences

Regional bone mineralization differences inside the animal's body could be demonstrated in this study (Tables 2 and 3). The head showed the highest BMD and BMC over all body regions and over all scans. BMD and BMC also differed between front and hind legs as well as between femur and lower hind legs. In this study, the front limb (R3/R4) was evaluated in total (shoulder blade to toe) and the hind leg was divided into femur (separated from the hip at the *caput femoris*) and lower hind leg (knee to toe), due to the identifiability of different bones in the hind limb (especially the hip). Ryan et al. (2011) published the finding that the BMD of the front limbs is significantly higher than the BMD of the hind limbs. In the present study the BMD of the total front limbs (R3/R4) is higher than the BMD of the lower hind legs (R8/R9), but not higher than the BMD of the femur (R6/R7) analysed separately. On the other hand, the highest BMC was found in the front limbs (R3/R4). This higher BMC could be explained by more weight loaded on the front limbs (due to the head and neck), as published by Mitchell et al. (2001). Additionally, as published by Klein-Nulend et al. (2005), an increased osteoblast activity due to weight load underlines this hypothesis. Thorup et al. (2007) showed that front limbs differ bio-mechanically, as they carry more weight and have higher peak forces and longer stance phases when walking. Although DXA is used as the method of choice for bone mineral determination in humans (Carter et al., 1992; Tothill and Hannan, 2002, 2007), there are limitations based on the two-dimensional character with misleading results comparing bones of different thicknesses and sizes (Carter et al., 1992). Larger bones may have a higher BMD than smaller bones due to the different sizes (Bouxsein and Seeman, 2009). This should be kept in mind as the present study shows the highest BMD for the femur by contrast with the results of Ryan et al. (2011) with the highest BMD in the front limbs. Table 3 represents BMD and BMC results depending on the body region summarized over all groups and scans. This table confirms differences in body regions, with the head having the highest BMD. It also shows that both body sides (left and right side) were evaluated equally as both sides receive the same superscript (Table 3).

BMD is affected by many factors, like muscle or fat mass, activity, calcium–phosphorus mineralization, or sexual hormones. In this study only male animals were examined; therefore more weight due to a higher muscle or fat mass and a more developed shoulder and head region (e.g. Dobrowolski et al., 1995) are supposed to have had an effect on bone mineralization. Additionally, special needs regarding the calcium : phosphorus ratio could influence BMD (Aiyangar et al., 2010; Létourneau-Montminy et al., 2010; Ryan et al., 2011), especially in males which show high muscle mass. Burr et al. (2002) showed that weight bearing and activity affect BMD. In this study, no activity scoring was performed, but it has to be kept in mind that no significant difference was found regarding weight and age of the different sex type groups. Further evaluation of these DXA images is needed to evaluate muscle and fat tissue in the defined body regions to be able to compare muscle and fat tissue data with BMD or BMC data of the same region.

### 4.2 Discussion sex differences

Sex differences were more pronounced in the 50 kg scan than in the 30 kg scan (Table 2). At 50 kg, EB and IB, both representing entire males at that time point, showed less BMD (R1) than SB. At 90 kg, this changed, as IB took a position between EB with the significantly lowest BMD and SB with the significantly highest BMD (R1). In the present study no significant difference was found between the different sex types regarding bone mineral tissue or soft lean tissue mass (Table 7), although SB showed the (significantly) highest fat tissue (%, kg) over all examinations (Table 7). The fact that SB in comparison with EB or IB showed the highest fat content in the carcass is confirmed by other studies (e.g. Fàbrega et al., 2010). Investigations of boar carcasses showed that the body composition of an intact boar is significantly different from that of barrows or gilts (Dobrowolski et al., 1995; Bauer, 2010). Further studies are needed which include also female pigs to evaluate differences based on the hormonal status. Additionally, studies have to be performed evaluating the effects of low BMD on leg health compared with high muscle mass in entire boars because EB had the significantly lowest BMD in the leg regions (R4, R6, R8, and R9) compared to castrated males (IB and SB; Table 2). This reduction in BMD might result in less bone conformation and be followed by more lame fattening pigs, which must be avoided due to animal health and for economic reasons.

Further studies are needed which also include female pigs to evaluate differences based on the hormonal status (Almeida et al., 2017). In agreement with findings in growing humans with higher BMD in girls or young women in comparison to boys or young men (Boot et al., 1997), Kogelmann et al. (2013) did not find significant differences for BMD between female and male pigs – also with a slight advantage for female pigs. According to Kranioti et al. (2019), human males reach peak bone mass combined with the highest BMD later than females do. Therefore, it seems plausible that young entire boars – as in our study – do not have higher BMD values than the castrated ones (IB or SB).

### 4.3 Discussion group differences

The three examination groups (I, II, and III) were raised in different seasons with different periods of daylight; the detected differences among groups might be traced back to seasonal effects due to temperature, light access, or group constellation. Additionally, no significant differences were detected regarding weight, age, and bone mineral tissue mass among the groups. Group I was raised with the shortest period of daylight (July to December: fall to winter during fattening), group II was raised with slightly increasing period of daylight (September to February: winter during fattening), and group III was raised with the longest period of daylight in this study (January to July: early summer during fattening; Table 1). At scans 50 and 90, group II showed significantly lower fat tissue (% and kg) than group I and III, although group I and II did not differ regarding soft lean tissue (kg) at 90 kg (Table 7).

An effect on bone mineral tissue could not be confirmed, although other studies reported differences in humans (Krølner, 1983; Rico et al., 1994). Rico et al. (1994) detected an increase in BMD during summer–fall and a decrease during winter–spring in healthy premenopausal women (assessed by DXA measurements). Additionally, Arens et al. (2007) reported that sheep had the lowest BMD in winter compared with the BMD records in spring and summer (measured using quantitative computed tomography). This might be an effect of daylight, which could not be displayed in an outdoor climate barn as used in this study. To be able to discuss daylight effects, an outdoor study has to be performed with animals of different sex types housed in different seasons outdoor (if possible).

At scan 50 and 90, group II showed the significantly lowest fat content (kg, %). At scan 90, soft lean tissue mass was highest in group I and II. The group differences in soft lean tissue are significant at scan 90. At scan 30 and scan 50, no significant differences among groups could be detected. Group I has the highest soft lean tissue, followed by group II and III. In further studies, this should be studied in more detail, as seasonal changes in feeding or activity pattern might have effects on carcass composition.

## 5 Conclusions

DXA showed differences in bone mineralization in pigs, especially pronounced for different body regions and among male sex types. For further studies, it is necessary to evaluate whether a lower or higher BMC or BMD affects the health of male fattening pigs, as has been published in human medicine, where a declining bone mineral density is a surrogate for declining bone strength (Ryan, 1997; Bonnick, 2007).

Since the present results showed differences among sex types, it is necessary to evaluate the importance of these differences for lameness prevalence in further studies, in particular, as boar fattening is named as one possible alternative after the ban on piglet castration without anaesthesia in 2021 in Germany. Additionally, it seems necessary to examine whether seasonal differences have an effect on bone health or bone strength and how it should be handled in fattening pigs. Perhaps, these differences can be influenced by light programmes, outdoor areas, or food additives in terms of calcium or phosphorus add-ons.

Recently published genome-wide association studies by Rothammer et al. (2014, 2017) showed that further candidate genes or gene regions were found for whole-body or regional bone mineral traits assessed by using DXA in pigs in vivo. Therefore, genomic analysis and additional BMC or BMD phenotypes can add information in terms of leg health and animal welfare in pigs.

## Data Availability

Data will be made available on reasonable request.
